# Are BET Inhibitors yet Promising Latency-Reversing Agents for HIV-1 Reactivation in AIDS Therapy?

**DOI:** 10.3390/v13061026

**Published:** 2021-05-29

**Authors:** Thanarat Salahong, Christian Schwartz, Rungroch Sungthong

**Affiliations:** 1 Department of Immunology, Faculty of Medicine Siriraj Hospital, Mahidol University, Bangkok 10700, Thailand; thanarat.sal@student.mahidol.ac.th; 2 Research Unit 7292, DHPI, IUT Louis Pasteur, University of Strasbourg, 67300 Schiltigheim, France; 3 Institute of Biodiversity, Animal Health and Comparative Medicine, College of Medical Veterinary and Life Sciences, University of Glasgow, Glasgow G61 1QH, UK; 4 Laboratory of Hydrology and Geochemistry of Strasbourg, University of Strasbourg, UMR 7517 CNRS/EOST, 67084 Strasbourg CEDEX, France

**Keywords:** HIV-1, latently HIV-1-infected cell, latency-reversing agent, BET protein, BRD2, BRD4, LRA, BETi, epigenetics, immune response

## Abstract

AIDS first emerged decades ago; however, its cure, i.e., eliminating all virus sources, is still unachievable. A critical burden of AIDS therapy is the evasive nature of HIV-1 in face of host immune responses, the so-called “latency.” Recently, a promising approach, the “Shock and Kill” strategy, was proposed to eliminate latently HIV-1-infected cell reservoirs. The “Shock and Kill” concept involves two crucial steps: HIV-1 reactivation from its latency stage using a latency-reversing agent (LRA) followed by host immune responses to destroy HIV-1-infected cells in combination with reinforced antiretroviral therapy to kill the progeny virus. Hence, a key challenge is to search for optimal LRAs. Looking at epigenetics of HIV-1 infection, researchers proved that some bromodomains and extra-terminal motif protein inhibitors (BETis) are able to reactivate HIV-1 from latency. However, to date, only a few BETis have shown HIV-1-reactivating functions, and none of them have yet been approved for clinical trial. In this review, we aim to demonstrate the epigenetic roles of BETis in HIV-1 infection and HIV-1-related immune responses. Possible future applications of BETis and their HIV-1-reactivating properties are summarized and discussed.

## 1. Introduction

Although antiretroviral therapy in AIDS patients reduces viremia, continuous administration of drugs is required, since HIV-1 gene transcription still occurs at a residual level in latently HIV-1-infected cell reservoirs, mainly in resting CD4^+^ T cells, in patients under combination antiretroviral therapy (cART) [1]. This residual transcription is associated with chronic immune activation and low-level inflammation, which support non-AIDS co-morbidities [2]. However, there is now compelling evidence that the composition of HIV-1 reservoir is heterogeneous [3]. Indeed, HIV-1 is latently established in various cell types such as hematopoietic stem cells, dendritic cells, microglial cells or in cells from the monocyte-macrophage lineage reviewed in [4,5,6]. Moreover, these cells localize in a variety of anatomical sites including tissues such as the blood, the brain, the gut-associated lymphoid tissue, the adipose tissue, the bone marrow, and the genital tract [7], making it difficult to clear all virus reservoirs.

A prerequisite to successfully eliminate reservoirs is to understand the molecular mechanisms implicated in the establishment and maintenance of HIV-1 latency. Understanding these mechanisms could help us to discover new target proteins in the viral cycle, which are not affected by cART [8]. Some of these molecular mechanisms have been identified. An important role for the cellular cofactor CTIP2 (Bcl11b) was shown in the establishment and the maintenance of HIV-1 post-integration latency in microglial cells [9,10,11]. CTIP2 works as a scaffold protein recruiting at least two different complexes in microglial cells. As part of a chromatin remodeling complex, CTIP2 is associated with the lysine demethylase LSD1, the histone deacetylases HDAC1 and HDAC2, and the histone methyltransferase SUV39H1 [9,12,13,14]. Moreover, CTIP2 is also involved in the control of the elongation process of gene transcription by inhibiting P-TEFb functions [15,16].

**Figure 1:**
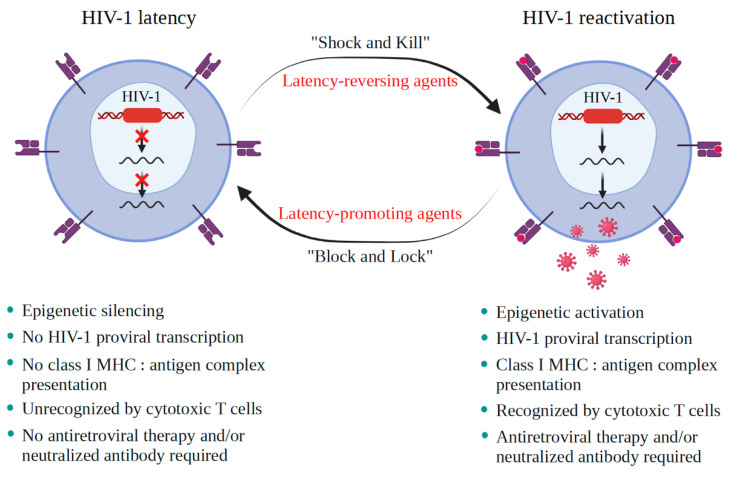
Concepts of “Shock and Kill” and “Block and Lock” strategies in AIDS therapy. Latency-reversing agents induce HIV-1 transcription via epigenetic activations in the “Shock” step. Then, these viral particles will be processed and released, leading to the elimination of the infected cells by immune clearance and to the elimination of the virus by combination antiretroviral therapy in the “Kill” step. Immune clearance involves CD8^+^ cytotoxic T cells (CLTs) that recognize the MHC I: HIV-1 peptide complex on the surface of HIV-1-infected cells and induce apoptosis by secreting granzyme B and perforin. An optimization of immunotherapy by using therapeutic vaccines to enhance CTL responses, broadly neutralizing antibodies and/or immune modulators are needed since viral reservoirs do not die from viral cytopathic effects or via the cytotoxic CTL responses. In the “Block and Lock” strategy, latency-promoting agents are applied for blocking the HIV-1 transcription in the “Block” step, and epigenetic silencing occurs in the “Lock” step. The figure was created with BioRender.com.

To date, two strategies are considered to achieve a functional and/or a sterilizing cure: the “Shock and Kill” and the “Block and Lock” strategies (Figure 1). The “Block and Lock” strategy aims to reach long-term control of HIV-1 in the absence of cART by inducing long lasting inhibition of HIV-1 gene expression [17]. Latency-promoting agents (LPAs) are molecules inhibiting HIV-1 expression, thus inducing deep latency (the “Block”) and preventing HIV-1 gene transcription (the “Lock”) [18]. The LPA, didehydro-cortistatin A (dCA), an inhibitor of the transactivator Tat, showed promising effect in preliminary results. However, a recent in vitro study described virus resistance to this factor [19]. Another recent pilot study, which assessed the effects of metformin on mTOR activation, showed reduced residual HIV-1 gene transcription in the gut-reservoir [20], suggesting that it could be a pertinent candidate among LPAs used in the “Block and Lock” strategy [21].

The “Shock and Kill” strategy proceeds by first reactivating the latent virus and subsequently eliminating it by a reinforced cART. Clearance of the reservoirs is achieved either by the cytopathic effect of the treatment on the reactivated virus and/or by inducing the immune system via the actions of cytotoxic T cells (CTLs) [22]. For the time being, the “Shock and Kill” strategy is only conceivable with circulating reservoirs such as the resting T CD4^+^ cells. Indeed, the “Shock and Kill” strategy cannot be applied when targeting brain reservoirs due to several unique characteristics of the central nervous system discussed in [10].

## 2. State of the Art of the “Shock” Step

The “Shock” step requires latency-reversing agents (LRAs), which play a crucial role in the reactivation of the virus as viral transcription activators. There are two main classes of drugs of LRAs under investigations.

The first class includes HDAC inhibitors (HDACis) such as valproic acid, vorinostat, panobinostat, romidepsin, and suberoylanilide hydroxamic acid (SAHA). For instance, SAHA activates viral transcription by inhibiting HDAC activity, which otherwise represses transcription [23,24]. In the first class, we find the histone methyl transferase inhibitors (HMTis) such as chaetocin and BIX 01294 and DNA methylation inhibitors such as 5-AzadC as well, as they are also involved in the epigenetic control of HIV-1. HDACis and HMTis have been revealed to reactivate to some extent HIV-1 expression both in vitro [25,26,27,28] and ex vivo [29]. However, among LRAs only HDACis (i.e., SAHA, panobinostat, romidepsin, and valproic acid) have been assessed in clinical trials [24]. Unfortunately, these compounds caused severe side effects such as anorexia, atrial fibrillation, diarrhea, exhaustion, and thrombocytopenia in AIDS patients [30]. 

The second class of LRAs comprises five subclasses of molecules: (i) non-histone chromatin modulators such as triazolothienodiazepine (JQ1) and BAF inhibitors; (ii) stimulators of positive cellular factors (NF-ĸB and the coactivator of NF-ĸB and P-TEFb) [31]. These drugs prompt the expression of positive cellular factors and/or their release from the inactive complex [32,33]. For example, prostratin, bryostatin, or ingenol B activates the PKC pathway and in consequence NF-ĸB and P-TEFb are released from the inactive complexes. Besides these actions, these agents also increase P-TEFb expression [34,35,36]. The active positive cellular factors in turn enable HIV-1 reactivation [37]; (iii) Toll-like receptor agonists; (iv) extracellular stimulators such as TNF α and PHA; v) miscellaneous drugs that comprise uncommon drugs such as disulfiram reviewed in [38].

However, these drugs used alone are ineffective to completely reactivate HIV-1 expression ex vivo [39,40]. This suggests that multifactorial mechanisms involving molecular reactions with underlying stochastic nature are responsible for the development of latency [3]. Thus, it appears that the feasibility of this strategy is rather difficult, mainly because of the poor comprehension of the molecular mechanisms involved in the establishment and maintenance of HIV-1 latency in reservoirs [41].

In latently HIV-1-infected cells, virus production is essentially blocked on the level of HIV-1 transcription. It is known that HIV-1 transcription is under epigenetic control of the HIV-1 promoter. Reactivation of transcription is inhibited directly by specific inhibitory mechanisms and/or by the sequestration of positive TFs. For instance, besides CTIP2 and LSD1, three new factors, Hic-1, HMGA1, and KAP1, have been involved in the repression of HIV-1 transcription in microglial cells [10,16,42]. These studies highlight the complexity of the molecular mechanisms underlying HIV-1 latency and explain why single LRAs failed in initial clinical trials. Post transcriptional events such as mRNA export, splicing and translation might also be essential in latency and deserve far more consideration [43,44].

To override these limitations, it was suggested that combination of drugs might improve the efficiency of reactivation along with reduced toxicity because of the synergetic effect and lower doses of drugs. Several clinical trials using the combinatorial approach are underway (reviewed in [38]). Next-generation LRAs are also in the focus of research. For instance, the inhibitors of bromodomains and extra-terminal domain (BET) family proteins, which are the master transcription elongation factors in epigenetic modification, are in the center of many current studies. BET inhibitors (BETis), such as JQ1 [45] and benzotriazolodiazepine (I-BET762) [46], initially were tested for their anticancer and anti-inflammatory effects, respectively. Research has been extended to evaluate the epigenetic roles and therapeutic potentials of BETis in other diseases (e.g., neurological disorders and obesity) [47], including AIDS [48,49]. A set of pioneer studies proved that JQ1 was a potential LRA candidate demonstrating strong reactivation of HIV-1 expression [48,49]. To date, there are growing numbers of synthesized BETis. However, their roles in epigenetics, HIV-1 reactivation, and host immune responses, together with their clinical perspectives in AIDS therapy, still need further investigation.

## 3. Roles of BET Family Proteins and Their Inhibitors in HIV-1-Infected Cells

Epigenetic modifications involve histones, chromatin-associated proteins, and the DNA. Proteins mediating these dynamic processes are the so-called “readers”, “writers”, and “erasers” [50,51]. Post-translational modifications of histones such as acetylation, methylation, sumoylation, phosphorylation, and ubiquitinylation, determine the compaction of chromatin and its permissiveness of transcription. Nuc-1, a nucleosome located immediately downstream of the HIV-1 transcriptional initiation site, is known to directly impede the HIV-1 promoter (the long-terminal repeat (LTR)) activity. Epigenetic modification and disruption of Nuc-1 was shown to be a prerequisite of activation of LTR-driven transcription and viral expression [52,53]. Deacetylases (erasers) are recruited by several transcription factors (TFs), including the homodimer p50, YY1, LSF or thyroid hormone receptors [53]. It was found that recruitment of erasers such as deacetylases and methylases on the LTR was linked to epigenetic modifications (deacetylation of H3K9 followed by H3K9 trimethylation and recruitment of HP1 proteins) in CD4^+^ T cells [54]. It was suggested that when the positive histone charge is neutralized by acetylation induced by the histone acetylase transferase (a writer), the chromatin structure is relaxed, and recognized by BET family proteins. These proteins have two tandem bromodomains (BRDs: BD1 and BD2) and one extra-terminal (ET) domain [55,56,57]. All BRDs have conserved sequences of ~110 amino acids, structurally consisting of four alpha-helices (αZ, αA, αB, and αC) and two loops (ZA and BC). The ET domain is a conserved region of ~80 amino acids. BET family proteins bind to the acetylated histone on their BRDs, allowing the ET domain to recruit TFs to form a transcription complex. Due to these functionalities, BET family proteins behave as epigenetic readers. The four members of BET family proteins are BRD2, BRD3, BRD4, and BRDT. 

### 3.1. BRD4

**Figure 2:**
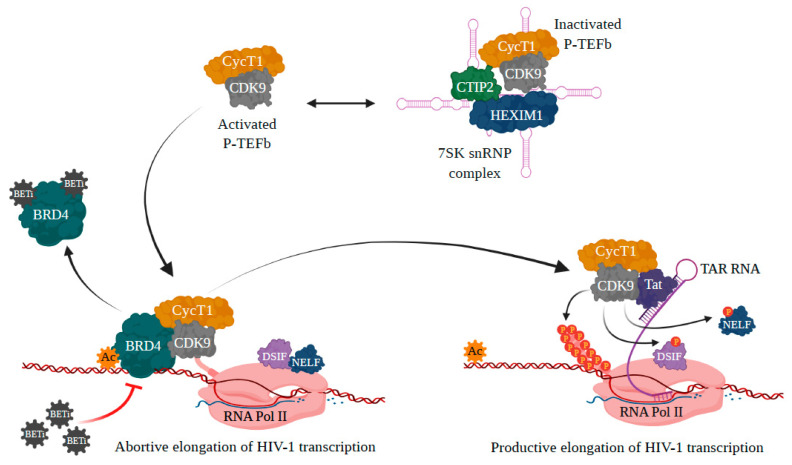
Roles of BET proteins and BETis in HIV-1 latency through the Tat-dependent manner. The 7SK snRNP forming complex which contains HEXIM1 and CTIP2 inactivates P-TEFb (CDK9 and CylinT1). During the cell activation, P-TEFb is released from the complex and becomes an active form that is the target for the viral Tat protein and BRD4. In HIV-1 latency, BRD4 competes with the viral Tat protein for recruiting active P-TEFb, leading to the low productive HIV-1 transcription. Thus, BRD4 inhibition by BETis increases the viral transcription via the phosphorylation of negative elongation factors (DSIF and NELF) and the serine 2 in CTD of RNA Pol II by the viral Tat/P-TEFb complex. The figure was created with BioRender.com.

The epigenetic reader protein BRD4 binds histones on acetylated sites. BRD4 as a scaffolding protein recruits many factors, e.g., NFκB and P-TEFb, which are involved in the regulation of gene expression [58,59]. Indeed, in addition to the conserved BRD and ET domain which characterize members of the BET family protein, BRD4 contains a third functional domain termed the P-TEFb-interacting domain (PID) [60]. BRD4 is described as a regulator of gene transcription elongation which occurs via its association with P-TEFb to form the super elongation complex (SEC) [61]. BRD4 recruits P-TEFb on the PID domain and releases it in its active state from the 7SK inactive complex, which also contains the 7SK RNA, HEXIM1, and CTIP2 inhibitors (Figure 2). Such inactive ribonucleoprotein complex associated with P-TEFb has been described in HeLa cells and other tumor cell lines [62,63]. However, in the case of HIV-1 transcription, Tat is a much stronger activator of P-TEFb than BRD4. Tat induces productive expression of HIV-1, while HIV-1 gene elongation is aborted in case of BRD4 stimulation. It is thought that BRD4 competes with the transactivator Tat, thus limiting HIV-1 transcription (Figure 2). It was shown that the BET inhibitor JQ1 blocked the interaction of BRD4 with the elongation factor P-TEFb composed of cyclin-dependent kinase 9 (CDK9) and cyclin T1 (CycT1). The results suggested that P-TEFb was free to associate with Tat, initiating productive HIV-1 elongation [64]. Once docked on the HIV-1 promoter, BRD4 induces autophosphorylation of CDK9 at threonine 29 inactivating CDK9 [59,65]. Another study [66] assumed that BRD4 interacted directly with the virulence factor Vif (viral infectivity factor) and CDK9 to form a ribonucleoprotein complex [67]. This interaction might allow cells to rest in the G_2_ phase, which supposes to enhance the establishment of HIV-1 latency.

Of note, the activity of JQ1 is essentially Tat-dependent but some studies reported that JQ1 induced activation of HIV-1 transcription in cell lines in the absence of Tat [68]. This might be explained by the existence of a short isoform of BET family member BRD4 (BRD4S), which acts as a corepressor of HIV-1 transcription. Indeed, the BRD4 isoform is bound to BRG1, a catalytic subunit of the BRG1-associated factors (BAF). BAF is a mammalian SWI/SNF chromatin-remodeling complex with known repressive functions in HIV-1 transcription [69].

Besides JQ1, other BETis are currently tested, such as OTX015, UMB-136, MMQO and I-BET151 [70]. Interestingly, OTX015 increases CDK9 occupancy at the HIV-1 promoter, which in turn phosphorylates RNA Pol II carboxy-terminal domain (CTD) [71], enabling productive elongation. 

### 3.2. BRD2

**Figure 3:**
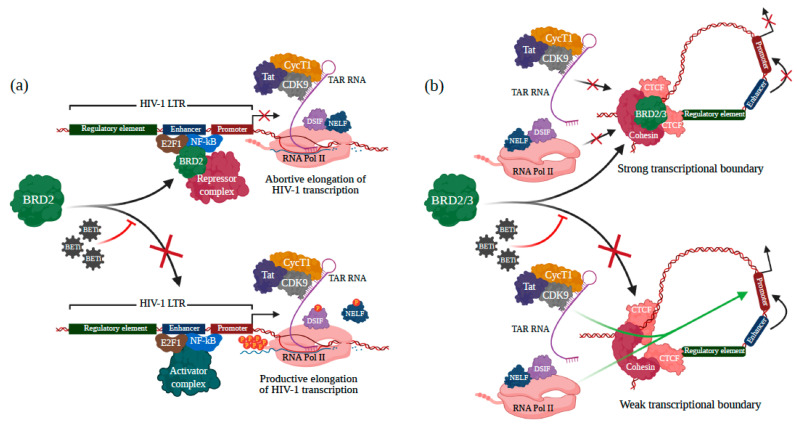
Roles of BET proteins and BETis in HIV-1 latency through the Tat-independent manner. (**a**) NF-κB and E2F1 initially recruit BRD2 to the viral LTR site (which consists of a regulatory element, an enhancer, and a promoter), and BRD2 recruits further the repressor complex by recognizing the acetylated lysine residue on such complex. The formation of the repressor complex represses HIV-1 transcription. When BETis inhibit BRD2, the activator complex binds to the viral LTR site, leading to the activation of viral transcription. (**b**) CTCF recruits BRD2 or BRD3 (BED2/3) to interact with the CTCF-cohesin complex and forms a transcriptional boundary that interrupts the viral transcription. BRD2/3 induces a strong transcriptional boundary that may modify the chromatin structure and interfere with the accessibility of HIV-1 transcription components. Hence, BRD2/3 inhibited by BETis weakens the transcriptional boundary and enhance the HIV-1 transcription by causing relaxed chromatin structure and facilitating the accessibility of transcription components to the viral genome site. The figure was created with BioRender.com.

In 1992, Beck and colleagues were the first to reveal the importance of BRD2 (formerly, a Really Interesting New Gene 3, RING3) in human developmental regulation mechanisms [72]. BRD2 is a major player in epigenetics. Along with BRD4, BRD2 belongs to the BET family but lacks a C-terminal PID domain. However, BRD2 was found to coimmunoprecipitate with P-TEFb [73]. A crucial role of BRD2 bound to acetylated histones is to activate P-TEFb (Figure 3a) [60]. Interestingly, BRD2 was found to function as a repressor of HIV-1 in latent cells. Indeed, depletion of BRD2 by shRNA activated HIV-1 transcription. In latently HIV-1-infected cells, the level of reactivation following knockdown of BRD2 was comparable to that of the BETi JQ1. In this case, reactivation of HIV-1 was dependent on P-TEFb activity but independent of the viral Tat [68]. It is believed that the mechanism of action involves co-repressor complexes including HDACs since BRD2 bound on an HDAC complex was observed [74]. In addition, BRD2 was shown to interact with the E2F1 TF. It was also shown that E2F1 was associated to the repressor NF-ĸB p50 on the HIV-1 promotor and blocked NF-ĸB p50/p65 dependent HIV-1 transcription [75]. In view of these results, it was postulated that BRD2 is recruited on the HIV-1 LTR by E2F1/p50 heterodimers while also enrolling repressor complexes which contain the erasers HDACs [76]. However, it is possible that another mechanism of action underlies repression of HIV-1 transcription by BRD2. It was shown that BRD2 associated with the architectural/insulator protein CCCTC-binding factor (CTCF), and they formed a transcriptional boundary [77]. Interestingly, CTCF was found associated to the HIV-1 promoter and prevented the formation of the NF-ĸB/LTR complex (Figure 3b) [78]. Bearing in mind the intricate epigenetic roles of BRD2, BETis may either hamper BRD2 recruiting the repressor complex (Figure 3a) or weaken the transcriptional boundary formation (Figure 3b). In both cases, virus transcription and reactivation should take place.

### 3.3. Other BET Family Proteins

It is not clear whether other BET family proteins such as BRD3 and BRDT are involved in the epigenetic regulation of HIV-1.

As described for BRD2 and BRD4, BRD3 is recruited onto the HIV-1 promotor via acetylated histones and permits the recruitment of TFs. BRD3 also provides support for interactions of epigenetic regulators to facilitate RNA Pol II performance [79,80]. The protein–protein interactions of BRD3 with other TFs/coactivators often regulate and activate the innate immune response [81]. For instance, a recent study revealed that BRD3 fostered the interferon regulatory factor IRF3/p300 complex recruitment to the *Ifnb1* promoter [82]. Like BRD2, BRD3 interacts with CTCF to form a transcriptional boundary [77,83]. Based on recent reports [77,83], we suppose that BRD3 induced HIV-1 latency, or its inhibitors induced reactivation, occurring via the transcriptional boundary-forming platform. However, the specific role of BRD3 in HIV-1 infection is still to be established.

With its definition “bromodomains specific-testis factor, BRDT” is typically silent in somatic and germ-line cells but has a crucial role in spermatogenesis and oogenesis at the onset of meiotic cell differentiation [84,85]. Moreover, in a study of a mouse model, it was unveiled that knockdown of the *Brdt* gene induces apoptosis during meiosis [84]. Although BRDT can interact with P-TEFb like BRD2 and BRD4, its uniquely affiliated cells are not the targets for HIV-1 infection.

## 4. BETis and Immunity

Although the epigenetic roles of BETis in HIV-1 latency and reactivation have been demonstrated and therefore are promising tools in the “Shock and Kill” strategy, further studies are needed to evaluate the consequences of their effects on the host immune responses. Here, we discuss how BETis affect the host immune system response to HIV-1 infection.

### 4.1. Innate Immunity

**Figure 4:**
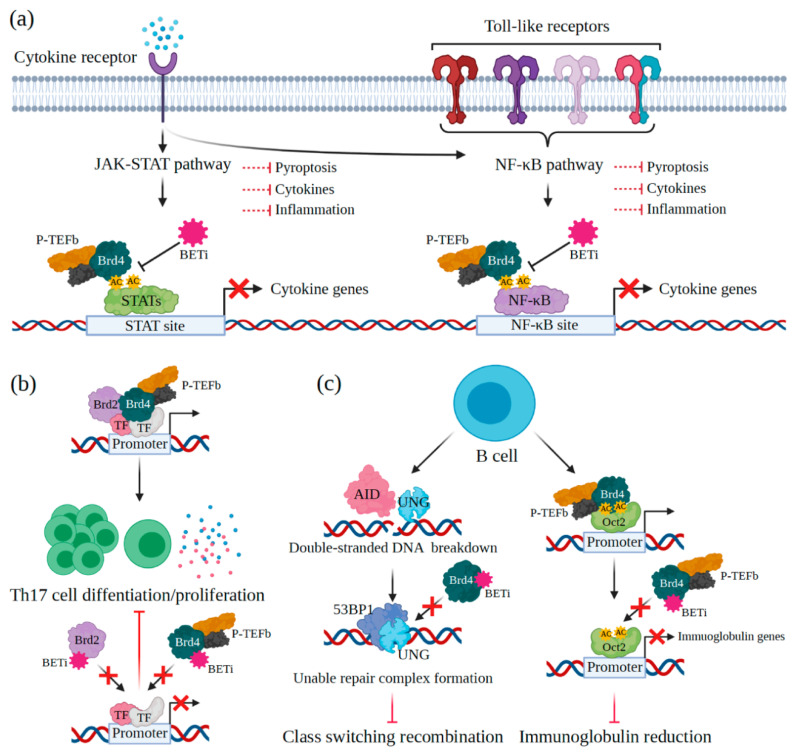
BETis and immune responses. (**a**) BRD4 recognizes an acetyl group on STATs and NF-κB, which are critical transcription factors in JAK-STAT and NF-κB signaling pathways, respectively. Once immune cells are exposed to BETis, BRD4 is then blocked, resulting in the decreased expression of cytokine gene. (**b**) BETis directly inhibit the functions of BRD2 and BRD4 mediating Th17 cell differentiation and proliferation in transcription regulation. (**c**) When BETis block BRD4, the repair complex is not formed, leading to the undeveloped CSR in B cells. Besides, after inhibiting BRD4, immunoglobulin genes are significantly suppressed, causing a low antibody level (AID is an activation-induced deaminase). The figure was created with BioRender.com.

Innate immunity is the first-line host defensive system to tackle pathogenic invasion. It involves diverse innate immune cells (e.g., dendritic cells, macrophages, NK cells), specific organs, and chemical molecules. The immune cells’ own pattern recognition receptors behave as host sensors for any invasive pathogens and host cell components released during cell damage and death [86,87,88]. Downstream signaling of the receptors is the keystone to prompt immune responses. BET proteins and especially BRD4 trigger the innate immunity through numerous downstream signaling pathways, such as Janus kinase-signal transducer and activator of transcription protein (JAK-STAT), NF-ĸB, or nucleotide-binding oligomerization domain-like receptor (NLR). Activation of these pathways promotes inflammation and pyroptosis (Figure 4a) [89]. During acute HIV-1 infection, diverse inflammatory cytokines are increasingly produced by innate immune cells, causing the cytokine storm, which is responsible for the high mortality rate in AIDS patients. At the same time, interleukin 6 (IL-6) and tumor necrosis factor-alpha (TNF-α) increase and are linked to the development of cardiovascular disease, atherosclerosis, and others [90,91,92,93]. Although antiretroviral therapy minimizes inflammation in AIDS patients, it is still higher than that of healthy ones [91,94,95,96,97,98]. Therefore, suppression of excessive cytokine production may improve AIDS therapy and patient immune responses. BETis have been proved for their capabilities to reduce IL-6, interferon gamma-induced protein 10 (IP-10), macrophage inflammatory protein 1 (MIP-1), and TNF-α and to prevent hyperinflammatory conditions during HIV-1 infection [99,100,101,102]. The inhibitors also suppress dendritic cell maturation, leading to the diminution of cytokine production [103,104]. Even though these benefits assure an important role for BETis in HIV-1 infection prompted hyperinflammation regulation, their long-term effects on the immune response need to be investigated in the future.

### 4.2. Adaptive Immunity

Another host defensive mechanism against invasive pathogens is adaptive immunity, and its key players are CD4^+^ T helper cells. CD4^+^ T helper cells are categorized based on the differences of cytokine production and surface marker expression [105,106]. T helper 1 (Th1) cells destroy intracellular pathogens by releasing interferon-gamma, IFN-γ being the signature cytokine. IFN-γ stimulates the adaptive and the innate immune systems, such as B cells, CTLs, classical macrophages, and NK cells. A recent study reported that a BETi (JQ1) suppressed IFN-γ expression in Th1 cells and memory CD4^+^ T cells [107]. Although IFN-γ is not a potent antiviral cytokine, circulating IFN-γ level is upregulated in AIDS patients [108,109]. It is still interesting how suppression of this cytokine affects immune responses during HIV-1 infection. Another subpopulation of CD4^+^ T helper cells is T helper 2 (Th2) cells that produce IL-4, IL-5, and IL-13 as defining cytokines [105,110]. Th2 cells can eradicate helminth infection through eosinophil stimulation, immunoglobulin E secretion, and mast cell activation. Additionally, Th2 cells are involved in tissue repair by promoting the alternatively activated macrophage (M2) polarization. A recent study [111] revealed that an elevated level of the chemokine (C-C motif) chemokine ligand 18 (an M2 macrophage marker) corresponded to a low number of CD4^+^ T cells in AIDS patients with antiretroviral therapy. Thus, immune responses of Th2 cells during HIV-1 infection are appealing to explore further.

In addition to Th1 and Th2 cells, T helper 17 (Th17) cells producing dominant cytokines (IL-17 and IL-22) can defeat invasive extracellular microbes at mucosal sites. However, Th17 cells are main targets of HIV-1 and make viral reservoir to persist, which leads to chronic inflammation [112,113]. These T helper cells highly express C-C chemokine receptor type 5 (CCR5) and minor receptors (C-C chemokine receptor type 6 (CCR6) and CXC chemokine receptor (CXCR) type 6). They facilitate infection by HIV-1 disrupting mucosal Th17 cell function and mucosal immune homeostasis. Antiretroviral therapy in AIDS patients cannot repair Th17 cell function [114]. One explanation is that increased indoleamine 2,3-dioxygenase 1 (IDO1) activity further induces regulatory T cell (Treg) expansion (i.e., inhibiting Th17 cell differentiation) by catabolizing tryptophan to kynurenine. Lately, the *IDO1* promoter is identified as a binding site for all BET family proteins (BRD2, BRD3, and BRD4) and the RNA Pol II [115]. Although some BETis (i.e., ABBV-075, JQ1, and OTX015) efficiently reduce the expression of both constitutive and IFN-γ-induced *IDO1* and kynurenine formation [115], their functions in mucosal Th17 cell repair are still unknown. Many studies proved that BETis could suppress Th17 cell differentiation/proliferation, cytokine production, and surface markers (Figure 4b) [116,117,118]. Therefore, Th17 cell recovery would be another preferable characteristic of potential LRAs for future AIDS therapy.

Treg cells, also known as “suppressor T cells”, are among the HIV-1 infected cells and responsible for the persistence of HIV-1 reservoirs [119]. Treg cells inhibit cell maturation of CTLs and in consequence presentation of B7-CD28 antigen. Moreover, Treg cells produce anti-inflammatory cytokines (i.e., IL-2, IL-10, and TGF-β) that further suppress other immune cells. Some studies found that Forkhead box-P3 (FOXP3, a master TF for regulating differentiation and suppressive functions of Treg cells) expression increased in AIDS patients and repressed HIV-1 transcription [120,121]. Previous studies [120,122] discovered that DNA methylation was altered during HIV-1 infection. Demethylation of the FOXP3 promoter was significantly higher in AIDS patients than in healthy ones, which led to the high FOXP3 expression and increased number of Treg cells in the gut mucosa. This observation suggests that HIV-1 may direct the replication of Treg cells by altering the FOXP3 methylation pattern. Although a BETi (JQ1) is proved to attenuate CD4^+^FOXP3^+^ Treg cell suppressive function in lung cancer therapy [123], how BET family proteins associate with FOXP3-promoting Treg cell functions is still unknown.

CTLs (or CD8^+^ T cells) eliminate HIV-1-infected cells after recognizing viral peptides presented on their major histocompatibility complex class I [124,125]. Studies suggested that BRD4 inhibited by BETis is related to the decreased CTL differentiation and proliferation [126,127,128]. However, the precise mode of interactions between BET family proteins and CTLs needs further elucidation. During chronic HIV-1 infection, CTL exhaustion is developed (i.e., reduced proliferation, decreased IFN-γ production, and insufficient cytotoxic activity), leading to defect HIV-1 clearance [129,130]. The programmed cell death protein 1 (PD-1) is a master inhibitory immune checkpoint molecule that interacts with the programmed death-ligand 1 (PD-L1) in CTL exhaustion development. Some BETis can interrupt the PD-1/PD-L1 signaling pathway in cancer treatments [131,132]. It would be interesting to examine how BETis affect CTL exhaustion during chronic HIV-1 infection. B cells are another key player in HIV-1 infection, that produce specific antibodies inactivating viral particles during the reinfection cycle [133,134,135]. B cell functions are also affected by BETis as BRD4 is one of the regulators in class switch recombination (CSR), a unique mechanism for altering specific B cells (Figure 4c) [136,137]. Inhibition of BRD4 leads to the reduced accumulation of 53BP1 (a p53-binding protein required in DNA repair) and uracil DNA glycosylase (UNG, generating DNA double-strand breaks) in the CSR process (Figure 4c) [136,137,138,139]. Besides, BETis block the interaction between Brd4 and Oct2 (an immunoglobulin promoter-binding TF), decreasing immunoglobulin production and B cell proliferation rate (Figure 4c) [140]. 

Although BETis are engaged in innate and adaptive immune responses, little is known about their molecular immunological influence on CTL development and functions. Besides, the impact of BETis on immune responses during HIV-1 infection still needs to be assessed using appropriate models in particular in vivo. Filling these knowledge gaps may underpin the implementation of BETis in future AIDS therapy.

## 5. BETis: Current and Beyond

Several BETis are investigated in clinical trials for their effectiveness in various diseases but not AIDS [141,142]. Although some studies unveiled HIV-1 latency-reversing activities of BETis, their implementations as LRAs in AIDS therapy are still far away. To our knowledge, I-BET-151 is the only BETi that was tested in vivo for its functions in HIV-1 reactivation [143]. Here, we describe some currently interesting BETis and their epigenetic roles relevant to HIV reactivation, impacts on host immune responses, and clinical perspectives (Table 1). We also address the limitations and discuss potential applications of BETis in AIDS therapy.

Apabetalone (RVX-208), CPI-203, I-BET-151, MMQO, OTX-015, PFI-1, and UMB-136 are interesting BETis that demonstrate similar HIV-1-reactivating mechanisms (Table 1). Some of these inhibitors were proved to inhibit BRD4, while some could block two or more BET family proteins (i.e., RVX-208, I-BET-151, OTX-015, and PFI-1) (Table 1). Studies of the BETis-BET family proteins interactions provide a comprehensive understanding of how viruses establish latency and how to process with optimal reactivation of the virus. Most BETis principally reactivate HIV-1 by inhibiting BRD4, which allows P-TEFb recruited by Tat on the viral promoter [71,144,145,147,151]. BETis also induce phosphorylation on threonine 186 of CDK9 and its subsequent activation and phosphorylation of serine 2 of the CTD of RNA Pol II, leading to the HIV-1 transcription elongation. On the other hand, the BRD4-induced autophosphorylation of CDK9 threonine 29 is known to silence HIV-1 transcription [59,65]. Hence, the link between BETi functions and CDK9 phosphorylation/dephosphorylation at threonine 29 and 186 would offer another means to control HIV reactivation.

Some BETis have shown their potential to modulate immune responses and prevent inflammation and the cytokine storm, mainly through BRD4 inhibition [153,154]. Chronic inflammation still occurs during HIV-1 infection, even under antiretroviral therapy, which causes T cell exhaustion [155,156]. Interestingly, several BETis (Table 1) suppress inflammatory cytokine productions in diverse HIV-1 latency models. It will be interesting to investigate how BETis prevent T cell exhaustion in HIV-1 latency and contribute to host immune responses in the “Kill” step. Furthermore, BETi treatments diminish the presence of HIV-1 receptors/coreceptors in primary CD4^+^ T cell membranes but do not alter the activation marker levels, suggesting that BETis neither globally activate immune cells nor support de novo HIV-1 infection (Table 1). Despite these promising features of BETis, which could advance the “Shock and Kill” strategy-based elimination of HIV-1, it is essential to confirm the effectiveness of BETis in in vivo and in clinical trials.

Li and colleagues [143] first revealed that I-BET-151 efficiently reactivated HIV-1-infected cells in humanized mice treated with antiretroviral drugs (Table 1). Surprisingly, this BETi only reversed HIV-1 latency in monocytic cells but not in CD4^+^ T cells, one of the principal targets of HIV-1. The authors suggested that CDK2 might disrupt I-BET-151 activity reactivating HIV-1 latency in CD4^+^ T cells, while CDK2 and CDK9 are required to promote HIV-1 transcription in monocytic cells. Another study unveiled that I-BET-151 could potentially reactivate HIV-1 from latent cell line models and primary CD4^+^ T cells [68]. These contradictory outcomes raise some keen questions about the role of CDK2 in HIV-1 latency and how it impacts HIV-1 transcription in CD4^+^ T cells. These findings emphasize the need for in vivo assessments of BETis. Some previous studies demonstrated that applying BETis with other LRAs (especially PKC agonists) offered greater HIV-1-reactivating potentials [68,144,145,147,150,151,152] (Table 1). Although PKC agonists induce cytokine production that can cause the cytokine storm, this problem can be overcome by the suppressive effects of BETis [147]. CPI-203 is the only BETi proved for its activity to suppress lipopolysaccharide (LPS)-stimulated inflammatory cytokines in vivo [147] but not in HIV-1-infected mice models (Table 1).

In addition to BETis listed in Table 1, MS402 and MT1 are BETis recently investigated for their cancer therapeutic potentials [157,158]. Although these inhibitors bind selectively to BRD4, their benefits as LRAs have not been proved. MS402 binds more selectively to BD1 than BD2 in BRD4 and prevents colitis in mice [157,159]. MT1 is an intramolecular bivalent BRD4 binder, offering higher efficacy than JQ1. Additionally, MT1 is successful in delaying leukemia progression [158]. It is worth checking out how these attractive BETis can be applied in HIV-1 reactivation and in AIDS therapy.

## 6. Concluding Remarks

The “Shock and Kill” strategy, which aims to reduce the pool reservoir, is based on the efficient reactivation of latent reservoirs, followed by the elimination of these reservoirs. Today, this strategy has shown its limits since both the “Shock” and the “Kill” steps do not optimally reduce the pool of reservoirs in vivo [38]. HDAC inhibitors used in the “Shock” step are the only LRAs currently tested in clinical trials but have shown multiple adverse effects in patients. The BETis, as transcriptional activators are promising LRAs that are worthwhile to test in AIDS therapy. Moreover, preliminary studies suggest that they have a positive impact on the immune system especially preventing inflammation and the cytokine storm. In vivo assessment and integrative application of BETis with other LRAs and antiretroviral drugs are the next steps required to evaluate their functions as HIV-1 LRAs.

## Figures and Tables

**Table 1:** Currently interesting BETis and their potentials in AIDS therapy.

BETi	HIV Model	Epigenetics and HIV Reactivation	Involved Host Immune Response	Clinical Perspectives
Apabetalone (RVX-208) [144,145,146]	In vitro ACH 2, J-Lat (A2, A10, C11, and 10.6), U1, and U937	• Binding selectively to BD2 in pan BET family proteins • Upregulating threonine 186 phosphorylation in CDK9 • Increasing Ser-2 phosphorylation in CTD of RNA Pol II • Facilitating Tat protein to recruit P-TEFb to the HIV-1 promoter by preventing BRD4 to bind the viral promoter	• Reducing CCR5 and CXCR4 surface expression on human primary CD4^+^ T cells • Unaffecting CD4, CD25, CD38, CD69, and HLA-DR surface expression on human primary CD4^+^ T cells • Unaffecting immune responses of CTLs and T helper cells • Unaffecting IL-2, IL-6, and TNF-α levels, but slightly increasing IFN-γ level • Inducing apoptosis	• Oral bioavailability • Immunosuppressive drug/chemokine inhibitor • Preventing de novo HIV-1 infection • Synergizing with prostratin and TNF-α • No additional side effect when tested in combination with other LRAs • Reactivating HIV-1 latency in vitro and ex vivo, and yet requiring in vivo tests
	Ex vivo Primary CD4^+^ T cells			
CPI-203 [147]	In vitro ACH 2 and J-Lat (A2 and 10.6)	• Binding selectively to BRD4 • Similar epigenetic roles and HIV-1 latency-reversing activities to RVX-208	• Reducing CD4, CCR5, and CXCR4 surface expression on human primary CD4^+^ T cells • Unaffecting CD25, CD38, CD69, and HLA-DR surface expression on human primary CD4^+^ T cells • Unaffecting IL-2, IL-6, TNF-α, and IFN-γ levels • Reducing LPS-mediated production of inflammatory cytokines (IL-1β, IL-6, and TNF-α) in THP-1 and a mouse model	• Oral bioavailability • Preventing de novo HIV-1 infection • Synergizing with prostratin and SAHA • Preventing cytokine storm when tested in combination with prostratin • Reactivating HIV-1 latency in vitro and ex vivo, and yet requiring in vivo tests
	Ex vivo Primary CD4^+^ T cells			
I-BET-151 [68,143]	In vitro J-Lat (A2 and A72)	• Binding to BET family proteins • Possibly reactivating HIV-1 latency by disassociating BRD4 from P-TEFb • Involving CDK2 and CDK9 regulation for HIV-1 transcription in monocytic cells and CD4^+^ T cells in vivo	• Undefined immune response in HIV-1 latency • Potentially suppressing inflammation and acting as immunosuppressive drug in other models [148,149]	• Unaffecting de novo infection in a mouse model • Synergizing with bryostatin-1 and prostratin • Non-cytotoxic to human myeloid and lymphoid cells in vivo during viral reactivation • Reactivating HIV-1 latency in vitro, ex vivo, and in vivo
	Ex vivo Primary CD4^+^ T cells			
	In vivo Humanized mice			
MMQO [150]	In vitro J-Lat (A2 and E89)	• Binding selectively to BD1 and BD2 in BRD4 • Dysregulating acetylation-sensitive genes • Undefined HIV-1 latency-reversing activity	• Robust immunosuppression • Undefined immune response in HIV-1 latency • Downregulating a set of genes in Jurkat cells	• Potentially new BETi class • Synergizing with prostratin, SAHA, and PMA • Reactivating HIV-1 latency in vitro and ex vivo, and yet requiring in vivo tests
	Ex vivo Primary CD4^+^ T cells			
OTX-015 [71,151]	In vitro J-Lat (A10 and C11)	• Binding to pan BET family proteins • Similar epigenetic roles and HIV-1 latency-reversing activities to RVX-208	• Unaffecting CD4, CD25, CD38, CD69, HLA-DR, CCR5, and CXCR4 surface expression on human primary CD4^+^ T cells • Reducing CD279 surface expression on CTLs • Unaffecting CD4^+^ T cell activation	• Potentially novel oral inhibitor • Preventing de novo HIV-1 infection • Synergizing with prostratin • No additional side effect when tested in combination with other LRAs • Non-cytotoxic to human primary CD4^+^ T cells • Reactivating HIV-1 latency in vitro and ex vivo, and yet requiring in vivo tests
	Ex vivo Primary CD4^+^ T cells			
PFI-1 [144]	In vitro J-Lat (A10 and C11)	• Binding to BRD2 and BRD4 • Similar epigenetic roles and HIV-1 latency-reversing activities to RVX-208	• Unaffecting CD4, CCR5, and CXCR4 surface expression on human primary CD4^+^ T cells • Unaffecting immune responses of CTLs and T helper cells	• Preventing de novo HIV-1 infection • Synergizing with prostratin and TNF-α • No additional side effect when tested in combination with other LRAs • Reactivating HIV-1 latency in vitro and ex vivo, and yet requiring in vivo tests
	Ex vivo Primary CD4^+^ T cells			
UMB-136 [152]	In vitro J-Lat (A2, 6.3, 8.4, 9.2, and 10.4)	• Binding selectively to BD1 in BRD4 • Undefined epigenetic role • Similar HIV-1 latency-reversing activities to RVX-208	• Unaffecting IL-2, IL-4, IL-6, IL-10, IL-17A, IFN-α, and IFN-γ levels	• Synergizing with bryostatin-1, prostratin, and SAHA • Reactivating HIV-1 latency in vitro and ex vivo, and yet requiring in vivo tests
	Ex vivo Primary CD4^+^ T cells			
